# Supplementary material for: Oleanolic Acid Derivative AXX-18 Exerts Antiviral Activity by Inhibiting the Expression of HSV-1 Viral Genes UL8 and UL52
Source: Viruses. 2022 Jun 13;14(6):1287. doi: 10.3390/v14061287 (PMC9227917; doi:10.3390/v14061287)
Supplement: Supplementary file 1 [file viruses-14-01287-s001.zip › viruses-1733671-supplementary.pdf]

Drug	CC50(μM)		EC50(μM)			
	Vero	HaCat	HSV-1/F	HSV-1/106	HSV-1/153	HSV-1/Blue
ACV	>50	>50	<0.5625	>20	>20	>20
AXX-18	39.38±0.7835	44.69±0.6916	1.47±0.3012	6.04±0.6032	9.29±0.5681	6.78±0.8062
OC	30.70±0.5673	36.20±0.9087	6.34±0.4059	12.74±1.0351	15.21±0.9073	12.96±0.7903

Table S1. The cytotoxicity and antiviral activity of AXX-18, OC, and ACV. CC_{50} is the 50% cellular cytotoxicity concentration. EC_{50} is the 50% effective concentration. Data are mean ± SD (n = 3).

The sequences of the target genes used in this study were obtained from the NCBI website, designed using Primer 5.0 software, and generated at Biotech Bioengineering (Shanghai) Co. The gene was dissolved in DEPC water at a concentration of 10 μM and stored at 20 °C.

Plasmid name	Primer sequence
pCMV-HA-UL8-F	CGGAATTCGGATGGACACCGCAGATATCGT
pCMV-HA-UL8-R	GGGGTACCATTTATTGGTCAAACTCAGGCA
pEGFPC1-UL52-F	CCAAGCTTCTATGGGGCAGGAAGACGGG
pEGFPC1-UL52 -R	CGGGATCCTCAAGACGACGGTTGAGAGGTG
p3Xflag-UL5-F	GCGGCCATGGCGGCGGCCGGCGGG
p3Xflag-UL5-R	AGGGATGCCACCCGGGATCCTTAATATACA

Table S2. Primer sequences for plasmid construction.

The target gene sequences used in this study were found on the NCBI website, designed on the website http://sidirect2.rnai.jp/, and generated at Biotech Bioengineering (Shanghai) Co. The gene was dissolved in DEPC water at a concentration of 20 μM and stored at 20 °C.

Target Name	siRNA Sequence
siUL5-1	Sense: GCAGCAACGTGATCGTCAT<br>Antisense: ATGACGATCACGTTGCTGC
siUL5-2	Sense: GGTTGTTCTCCTCCCACAA<br>Antisense: TTGTGGGAGGAGAACAACC
siUL8-1	Sense: GGGACTGGTGGTGAAAGTT<br>Antisense: AACTTTCACCACCAGTCCC
siUL8-2	Sense: GCGCGAATACCAGACTCTT<br>Antisense: AAGAGTCTGGTATTCGCGC

siUL52-1	Sense: GGAGCAAGACAGGTTCGAA<br>Antisense: TTCGAACCTGTCTTGCTCC
siUL52-2	Sense: CCATGTTCGTCTGTCGCTT<br>Antisense: AAGCGACAGACGAACATGG

Table S3. siRNA sequences.

The CDS sequences of the target genes in this study were obtained from the NCBI website, designed online using the NCBI website Primer BLAST, and generated at Biotech Bioengineering (Shanghai) Co. The gene was dissolved in DEPC water at a concentration of 10 µM and stored at 20 °C.

Gene Name	Sequence
α0-F	CCCACTATCAGGTACACCAGC
α0-R	CTGCGCTGCGACACCTTTT
α4-F	CGTGGTGGTGCTGTACTCG
α4-R	GAGCAGCCCCAGAAACTCC
α22-F	TACGCTGGAAACCCCAGAAC
α22-R	TCCAGACACTTGCGGTCTTC
α27-F	TGGCGGACATTAAGGACATTG
α27-R	TGGCCGTCAACTCGCAGA
α47-F	ATAAAAGGGGGCGTGAGGAC
α47-F	GATGTGCCACACCCAAGGAT
UL47-F	TACGAGGAGGACGACTACCC
UL47-R	ATCCGGACACGGGTAAAACC
UL5-F	GCACGAGTTCGGTAACCTCA
UL5-R	ACTCCTTGACCGACACGAAC
UL8-F	TCCGGTGGTGATGTTAACGG
UL8-R	GCAGATATCGTGTGGGTGGA
UL52-F	AATACGGCGCTCCACGTAAA
UL52-R	CTCATACACCAGGTCACGCT
GAPDH (human)-F	CACCATCTTCCAGGAGCGAG
GAPDH (human)-R	AGAGGGGGCAGAGATGATGA

Table S4. RT-qPCR primer sequences.
